# Circulating Tumor Cell Composition in Renal Cell Carcinoma

**DOI:** 10.1371/journal.pone.0153018

**Published:** 2016-04-21

**Authors:** Ivonne Nel, Thomas C. Gauler, Kira Bublitz, Lazaros Lazaridis, André Goergens, Bernd Giebel, Martin Schuler, Andreas-Claudius Hoffmann

**Affiliations:** 1 Molecular Oncology Risk-Profile Evaluation, Department of Medical Oncology, West German Cancer Center, University Duisburg-Essen, Essen, Germany; 2 ABA GmbH & Co. KG, BMZ2, Dortmund, Germany; 3 Department of Radiotherapy, University of Duisburg-Essen, Essen, Germany; 4 Institute for Transfusion Medicine, University Hospital Essen, University Duisburg-Essen, Essen, Germany; 5 Department of Medical Oncology, West German Cancer Center, University Duisburg-Essen, Essen, Germany; Chang Gung University, TAIWAN

## Abstract

**Purpose:**

Due to their minimal-invasive yet potentially current character circulating tumor cells (CTC) might be useful as a “liquid biopsy” in solid tumors. However, successful application in metastatic renal cell carcinoma (mRCC) has been very limited so far. High plasticity and heterogeneity of CTC morphology challenges currently available enrichment and detection techniques with EpCAM as the usual surface marker being underrepresented in mRCC. We recently described a method that enables us to identify and characterize non-hematopoietic cells in the peripheral blood stream with varying characteristics and define CTC subgroups that distinctly associate to clinical parameters. With this pilot study we wanted to scrutinize feasibility of this approach and its potential usage in clinical studies.

**Experimental Design:**

Peripheral blood was drawn from 14 consecutive mRCC patients at the West German Cancer Center and CTC profiles were analyzed by Multi-Parameter Immunofluorescence Microscopy (MPIM). Additionally angiogenesis-related genes were measured by quantitative RT-PCR analysis.

**Results:**

We detected CTC with epithelial, mesenchymal, stem cell-like or mixed-cell characteristics at different time-points during anti-angiogenic therapy. The presence and quantity of N-cadherin-positive or CD133-positive CTC was associated with inferior PFS. There was an inverse correlation between high expression of *HIF1A*, *VEGFA*, *VEGFR* and *FGFR* and the presence of N-cadherin-positive and CD133-positive CTC.

**Conclusions:**

Patients with mRCC exhibit distinct CTC profiles that may implicate differences in therapeutic outcome. Prospective evaluation of phenotypic and genetic CTC profiling as prognostic and predictive biomarker in mRCC is warranted.

## Introduction

Circulating tumor cells (CTC) are thought to be useful in individualizing and monitoring treatment in patients with solid tumors [1,2]. So far, CTC detection methods consist of enrichment and subsequent identification mostly with anti-cytokeratin (CK) or epithelial cell adhesion molecule (EpCAM) antibodies [3]. The epithelial-to-mesenchymal transition (EMT) can cause alteration of cellular features and loss of epithelial properties leading to a partial or complete switch to a mesenchymal phenotype. Particularly stem cells have the ability to take on characteristics of other cell types [4]. Yu and colleagues reported that the process of EMT is reversible during systemic treatment and that each cycle of response and progression associates with switches in the phenotype of CTC [5]. Since metastatic renal cell carcinoma (mRCC) cells often lack epithelial differentiation and currently available enrichment and detection techniques are often challenged by the cellular heterogeneity and plasticity of CTC, only a few reports have been published on the isolation of CTC in metastatic renal cell carcinoma mRCC [6–8].

We recently developed a CTC detection method based on multi-parameter immunofluorescence microscopy (MPIM) that includes epithelial markers such as CK or EpCAM and cells with mesenchymal and stem cell-like characteristics. We were able to identify an individual composition of CTC subtypes as profiles that associate to therapeutic success in hepatocellular carcinoma, non-small cell lung carcinoma and head and neck squamous carcinoma [9–13]. In this study, we have addressed the question whether different types of CTC are identifiable in the peripheral blood of patients with mRCC and, if so, whether their distribution may serve as a predictor of treatment response or outcome. Furthermore, we have assessed whether the distribution of these cells correlated to inter-individual differences in the expression of angiogenesis related molecular markers.

In renal cell carcinoma increased understanding of genetics and molecular biology led to successful employment of agents targeting the VEGF and mTOR pathways [6]. The resulting plurality of available treatment options is significantly limited by available parameters for a personalized implementation of these agents. We therefore tested CTC profiles together with gene expression levels of several candidate markers involved in angiogenesis like vascular endothelial growth factor A (*VEGFA)*; vascular endothelial growth factor receptor (*KDR1* also known as *VEGFR*); basic fibroblast growth factor (*FGF2)*; basic fibroblast growth factor receptor 1 (*FGFR1)*; platelet-derived growth factor alpha (*PDGFA)* and hypoxia inducible factor 1 alpha (*HIF1A*) for their association with response to first-line VEGF-targeted therapy. We then evaluated the association of the individual CTC composition with these molecular markers and their combined correlation to treatment outcome. With this approach we wanted to scrutinize whether developing a blood-based multi-marker panel for personalized treatment of mRCC is warranted.

## Materials and Methods

### Ethic statement and study population

Written informed consent was obtained from all patients before participating in the study. Blood sample collection and analyses were approved by the Review Board of the Medical Department, University of Essen-Duisburg; Germany (12-5047-BO). Prior to application of systemic anti-angiogenesis treatment 12 out of 14 patients had undergone nephrectomy during their history of disease. The clinical-pathological characteristics of the patients are listed in Table 1. Tumor staging was performed according to the criteria of the American Joint Committee of Cancer (AJCC) [14]. Response Evaluation Criteria in Solid Tumors (RECIST 1.1) were used to define response or stable disease in patients after receiving 2 cycles of systemic anti-angiogenesis therapy [15].

**Table 1:** Patient Demographics.

	Patients (n = 14)	
Demographic	No.	%
Tumor stage		
IV	14	100
Histo		
clear cell	12	86
non-clear cell	1	7
papillary	1	7
Grading		
G1	2	14
G2	3	21
G3	7	50
Gx	2	14
Age		
Median, years	61	
Range	38–78	
Heng Score		
good	2	14
intermediate	9	64
poor	3	21
Therapy		
Sunitinib	9	64
Pazopanib	3	21
Temsirolimus	2	14
Response		
PR	5	36
SD	9	64
PFS (months)		
Median	12	
Range	3–32	

Abbreviations: SD, stable disease; PR, partial response; G1: well differentiated; G2: moderately differentiated; G3: poorly differentiated; Gx: Grade cannot be assessed

### Preparation of cell lines

HepG2 cells were purchased from Sigma Aldrich (St. Louis, MO) and used within 6 months after resuscitation. DNA profile was characterized by the cell bank using Short Tandem Repeat (STR)-PCR. Gastrointestinal stromal cells (GIST 882) were a kind gift from Sebastian Bauer (Department of Medical Oncology, University Hospital Essen) and Jonathan A. Fletcher (Department of Pathology, Brigham and Women's Hospital/Harvard Medical School, Boston, MA). GIST882 cells expressed a c-KIT allele with an exon 13 missense mutation, resulting in a single amino acid substitution, K642E [16] which was frequently tested using DNA sequencing.

The stably CD133-expressing K562 cell line K562-CD133:IEG was generated by lentiviral-mediated gene transfer of a CD133/Prominin-1 splice variant s1 (GenBank accession number AF507034) cDNA–IRES-eGFP expression cassette [17]. Briefly, respective lentiviral supernatant was produced by cotransfection of HEK293T cells with the plasmids pCL1-CD133-IEG, pCD/NL-BH and pcoPE [18–20] using Jetpei (Polyplus, Illkirch Cedex, France) transfection reagent. At 16 h post transfection, cells were cultured for 6–8 h with sodium butyrate (10 mM, Sigma-Aldrich), and viral supernatant was collected after 48 h. K562 cells were transduced with lentiviral supernatants. The established cell line was passaged at least 10 times before experiments. K562 cells originally were obtained from ATCC and regularly tested in NK cell killing assays and by flowcytometric immunophenotyping. The cell line K562-CD133:IEG was characterized to express extracellular CD133 by flowcytometric stainings using PE-conjugated anti-CD133 antibodies (clone AC133; Miltenyi Biotec, Bergisch Gladbach, Germany; S1 Fig).

### Preparation of blood samples and CTC enrichment

**Fig 1:**
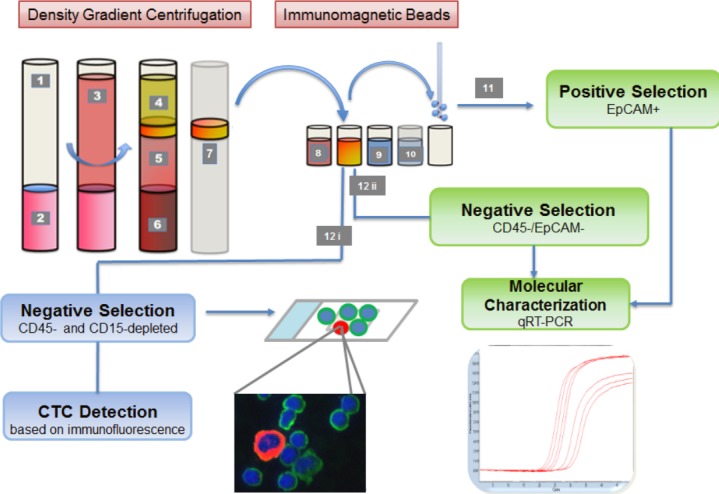
Basic principle of CTC isolation. 1. Leucosep tube: 2. Separation media; 3. Whole blood/PBMNC mixture; 4. Plasma; 5. Separation media after centrifugation; 6. Erythocytes; 7. Buffy coat incl. CTCs; 8. Anti-CD45 beads; 9. Anti-EpCAM beads (positive isolation) or anti-CD15 beads (negative isolation); 10. Washing buffer; 11. EpCAM-bead bound CTC suspension for qRT-PCR; 12i. Depleted bead free cell suspension containing CTCs for Cellspin and immunofluorescence staining. 12 ii. Depleted bead free cell suspension containing CD45-/EpCAM- CTC for qRT-PCR.

Duplicates of 20 ml citrated peripheral venous blood were drawn from mRCC patients after response assessment (2 cycles of therapy) and processed within 24 h after collection. Blood sample preparation was done as described previously [9–12]. Briefly, 20 ml of blood were diluted with 10 ml PBS and carefully layered into a Leucosep tube containing 16 ml Ficoll-Paque (GE-Healthcare) below a porous barrier. After buoyant density gradient centrifugation (1600 x g, 20°C, 20 min) the interphase consisting of peripheral blood mononuclear cells (PBMNC) and CTC was removed and washed. We employed an enrichment strategy consisting of 2 steps (Fig 1). For (i) subtype analyses CTC were negatively enriched by hematopoietic cell depletion. PBMNC were treated with 50 μl of a 1:1 mixture of anti-CD45 and anti-CD15 coated immunomagnetic beads (Dynabeads, Invitrogen) in a magnetic particle processor (King Fisher mL; Thermo Fisher, Waltham, USA). The remaining cell suspension included bead-free pre-enriched tumor cells and was spun onto two glass slides per sample using the Cell Spin II centrifuge (Tharmac, Waldsolms, Germany), air-dried and subsequently fixated with 96% Ethanol. Slides were stored at 4°C until subjected to immunocytochemical staining. For (ii) gene expression analysis a second sample was prepared in the same manner, but epithelial CTC were further enriched using anti-EpCAM immunomagnetic beads (Epithelial Enrich, Dynabeads, Life Technologies, Carlsbad, USA) resulting in an EpCAM-positive CTC suspension for molecular analysis.

### Identification of CTC subtypes using MPIM

Immunofluorescence staining of epithelial, mesenchymal, stem cell-like and hematopoietic cells was carried out in the CD45-depleted pre-enriched tumor cell suspension as described previously [9–11]. Briefly, the staining method included fixation of the cells in 4.5% paraformaldehyde for 15 min, washing in PBS, permeabilization with 1x Perm/Wash Buffer (BD Biosciences, Franklin Lakes, USA) for 10 min, washing in PBS, blocking of unspecific antibody reactions by incubation with blocking solution containing 5% BSA for 30 min, binding of primary antibodies (final concentration: 5 μg/ml) either anti-pan-CK guinea pig polyclonal antibody (ABIN126062, antibodies-online, Atlanta, GA) and anti-N-cadherin (EPR1792Y) rabbit monoclonal antibody (2019–1, Epitomics, Burlingame, CA) or anti-CD133 rabbit polyclonal antibody (orb18124, biorbyt, Cambridge, UK) for CTC and anti-CD45 (MEM-28) mouse monoclonal antibody (ab8216, Abcam, Cambridge, UK) for hematologic cells overnight at 4°C, wash in 0,1% Tween, binding of secondary antibodies (FITC-conjugated AffiniPure goat anti-rabbit and Cy3-conjugated AffiniPure goat anti-mouse or AlexaFlour647-conjugated AffiniPure F(ab’)2 Fragment goat anti-guinea pig; Jackson Immuno Research, Hamburg, Germany) for 30 min at 37°C, washing in 0,1% Tween. Subsequently, cells were stained with 4’6-Diamidino-2-phenylindole dihydrochloride (DAPI; Sigma-Aldrich, St. Louis, MO) for 10 min, mounted with anti-fading medium (Invitrogen) and stored in the dark until evaluation. As described previously, for each test a control slide with a mixture of PBMNC (CD45-positive, pan-CK-negative) from a healthy donor spiked with epithelial cells from the hepatocellular carcinoma cell line HepG2 (CD45-negative, pan-CK-positive) was treated under the same conditions. GIST 882 cells were used as positive control for the mesenchymal marker N-cadherin. Stably transduced CD133-expressing K562 cells (K562-CD133:IEG) were used as positive control for stem cell marker CD133. Microscopic evaluation was carried out using the digital Keyence BZ9000 (Biorevo, Osaka, Japan) all-in-one fluorescence microscope with integrated camera and BZ-Analyzer Software. We used pseudo colors to depict cells. Stained slides were manually examined and CTC were detected within the same areas, each consisting of 10 visual fields using a 20x magnification on both slides. Samples from 5 healthy donors were processed and examined under the same conditions in order to define cut-off values for false-positive events.

### RNA extraction and quantitative RT-PCR

For gene expression analyses epithelial CTC were sequentially enriched by depletion of hematopoietic cells and subsequent positive selection using anti-EpCAM immunomagnetic beads as described above [12]. Total RNA was extracted from recovered EpCAM-positive tumor cells using MagAttract RNA Cell Mini M48 Kits (Qiagen, Hilden, Germany) and King Fisher mL magnetic particle processor (Thermo Fisher, Waltham, MA) according to the manufacturer’s instructions. Additionally, remaining DNA was removed using RQ1 RNase free DNase (Promega, Fichtburg, WI). One-step real time RT-PCR (Roche LightCycler 480, Basel, Switzerland) was performed using Precision OneStep qRT-PCR Mastermix Kit with SYBR Green (Primerdesign, Southampton, UK) for gene expression analysis of *VEGFA; KDR1 (VEGFR) FGF2; FGFR1; PDGFA* and *HIF1A* (Primerdesign, Southampton, UK). The primers for reference gene beta-actin (Eurofins MWG, Nantes, France) were as follows: forward: 5’-GAGCGCGGCTACAGCTT-3’, reverse: 5’-TCCTTAATGTCACGCACGATTT-3’. Assays were performed in triplicates to determine expression levels. Thermal cycling conditions were 10 min at 50°C and 5 min at 95°C for RT and initial denaturation followed by 50 cycles of 95°C for 10 sec and 60°C for 30 sec. Triplicates of A549-RNA (10ng/μl) were used as internal standard to control each run. Each primer was validated in a serial dilution of RNA extracted from the cell line mentioned above.

### Statistical analysis

Statistical tests were performed according to previously published studies by our group [9–12,21,22]. Recursive descent partition analysis was used to identify the strongest divisor of all factors and the most significant split determined by the largest likelihood-ratio chi-square statistic in relation to clinical response as described previously [22,23]. The associations among CTC subtypes, gene expression levels and clinical-pathological parameters were tested with Spearman test for bivariate correlations. Mann-Whitney test for independent samples was used to compare differences of various factors in distinct subgroups. For gene expression analysis we used Wilcoxon signed rank test to assess whether expression levels differ in cell fractions after depletion of hematopoietic cells and enrichment of epithelial CTC. To identify potential independent factors associated with response multivariate regression models along with established clinical parameters were used. The Kaplan-Meier method was used to test correlations of PFS with cell types and gene expressions, respectively. Survival differences between patients with a high and low cell type ratio were analyzed by the log-rank test. The level of significance was set to P<0.05. All P values were based on two-sided tests. All statistical analyses were performed using the Software Packages JMP 10.0 Software (SAS, Cary, NC, USA), SPSS for Windows (Version 19.0; SPSS Inc., Chicago, IL) and Medcalc, Version 12.3.0 (Mariakerke, Belgium).

## Results

### Immunofluorescence based identification of CTC subtypes

**Fig 2:**
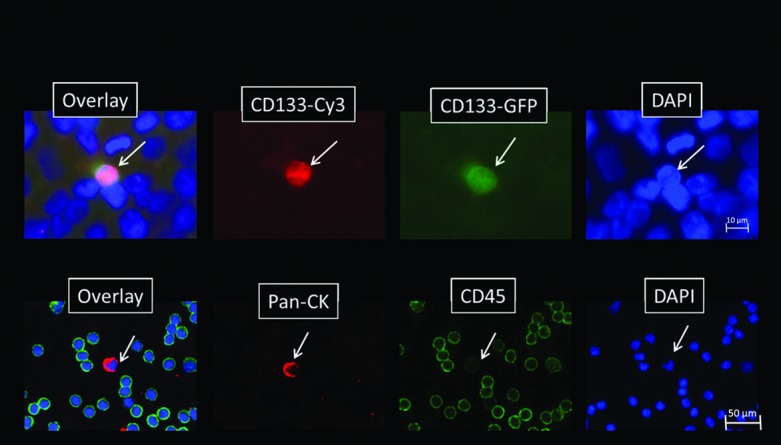
CTC detection. A) Positive control consisting of PBMNC mixed with CD133-expressing K562 cells which were stably transduced with lentiviral vectors endocoding an internal ribosomal entry site (IRES)-mediated co-expression cassette of CD133 and enhanced green fluorescent protein (eGFP) (K562-CD133:IEG). Cells were stained with DAPI (nucleus; blue) and for CD133 (pseudo-color red). The antibodies used for anti-CD133 immunostaining specifically bound to K562-CD133:IEG cells, visualized by Cy3 (red) counterstaining (40x magnification). B) CTC isolated from mRCC patients stained with DAPI (blue), for pan-CK (epithelial; red) and for CD45 (hematopoietic; green; 20x magnification). The cell marked with a white arrow shows a DAPI-positive (blue)/CD45-negative staining and was positive for pan-CK (red) and subsequently considered as CTC.

**Fig 3:**
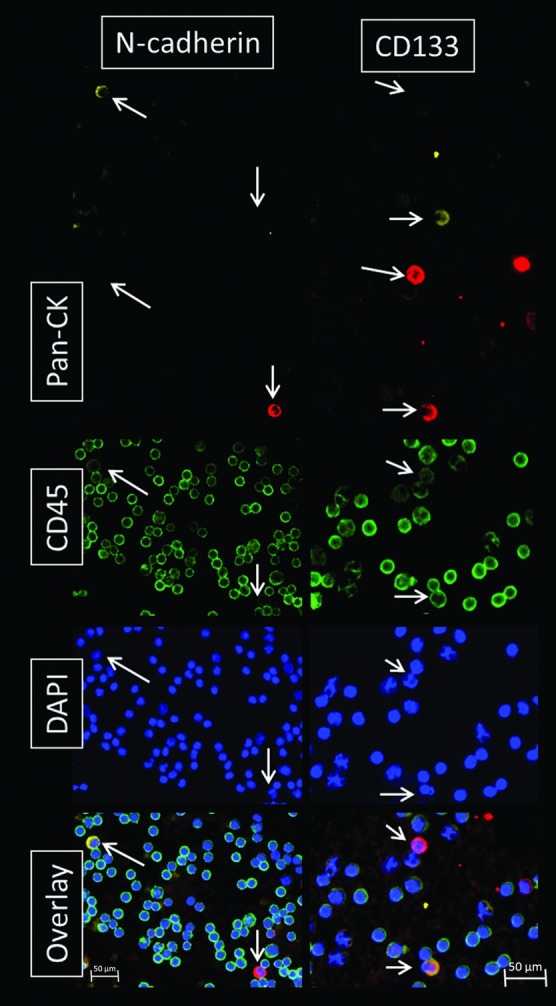
Detection of CTC subtypes. CTC isolated from mRCC patients were stained with DAPI (nucleus; blue) and for CD45 (hematopoietic; green), pan-CK (epithelial; red) and N-cadherin (mesenchymal; yellow) on one slide and with DAPI and for CD45, pan-CK and CD133 (stem cell; yellow) on a second slide. Cells marked with a white arrow were considered as CTC. The image displays various CTC subtypes with epithelial, mesenchymal and/or stem cell-like features such as N-cadherin+/CK-/CD45-; N-cadherin-/CK+/CD45-; CD133+/CK+/CD45+ and CD133-/CK+/CD45 (low) cells.

For the investigation of cellular subtypes a multi-staining method was required in order to detect various epithelial, mesenchymal, stem cell-like and hematopoietic characteristics. Therefore, we used MPIM for CTC-subtype detection in mRCC patients. Hematopoietic K562 cells stably transduced with a CD133-IRES-eGFP expression cassette and GIST 882 cells were used as controls for stem cell (CD133, Fig 2 Row A) and mesenchymal (N-cadherin) marker expressions, respectively. Cells from patient samples that showed a positive nuclear staining with DAPI, a negative staining for CD45 and a positive staining for pan-CK, N-cadherin or CD133 were captured and considered as tumor cells (Fig 2 Row B). In mRCC blood samples we detected cells with mesenchymal features such as N-cadherin+/CK-/CD45- and cells with epithelial properties like CK+/N-cadherin-/CD45- and cells with both characteristics like CK+/N-cadherin+ (Fig 3). We also detected cells showing both, stem cell-like and epithelial features such as CD133+/CK+ cells. CTC were enumerated and CTC profiles of each patient were examined. We scored the total amount of N-cadherin-positive, CK-positive and CD133-positive cells, and calculated a ratio of mesenchymal to epithelial cells after negative enrichment using CD45-depletion. We normalized the enumerated potential CTC against the total PBMNC number detected in the DAPI channel in each visual field and calculated the number of CTC per 1000 PBMNC. Analysis of samples from healthy donors revealed the following cut-off values for false positive events per 1000 PBMNC after CD45 depletion: 0.1 CD133+/CK+ cells; 0.1 CD133/CK- cells; 0.03 N-cadherin+/CK+ cells; 0.05 N-cadherin+/CK- cells and a total of 0.6 CK+ cells (Table 2).

**Table 2:** CTC quantification.

**Healthy donors**						
**Subgroup**	**No. of positive**	**Amount of cells/1000 PBMNC after enrichment**				
** **	**samples (n)**	**Minimum**	**Maximum**	**Mean**	**SD**	**cut-off**
CK+	12	0.09	0.57	0.19		0.6
CD133+						
CD133+/CK+	6	0.05	0.11	0.02	0.04	0.1
CD133+/CK-	3	0.03	0.11	0.02	0.03	0.1
N-cadherin+						
N-cadherin+/CK+	2	0.02	0.03	0.02	0.01	0.03
N-Cadherin+/CK-	1		0.05	0.05	0.01	0.05
						
**mRCC patients**						
**Subgroup**	**No. of positive**	**Amount of cells/1000 PBMNC after enrichment**				
** **	**samples (n)**	**Minimum**	**Maximum**	**Mean**	**SD**	** **
CK+	9	2.0	23.5	7.2	7.4	
CD133+						
CD133+/CK+	5	0.4	6.9	2.7	2.3	
CD133+/CK-	4	0.4	2.2	1.2	0.8	
N-cadherin+						
N-cadherin+/CK+	4	0.5	7.3	2.9	3.1	
N-Cadherin+/CK-	5	0.7	1.2	0.6	0.3	

Abbreviations: CK+: Pan-Cytokeratin-positive cells; CD133+: CD13- positive cells; N-cadherin+:N-cadherin-positive cells; CD133+/CK+: CD133-positive and Pan-Cytokeratin-positive cells; CD133+/CK-: CD133-positive and Pan-Cytokeratin-negative cells; N-cadherin+/CK+: N-cadherin-positive and Pan-Cytokeratin-positive cells; N-cadherin+/CK-: N-cadherin-positive and Pan-Cytokeratin-negative cells

### CTC-subtypes and clinical outcome

**Fig 4:**
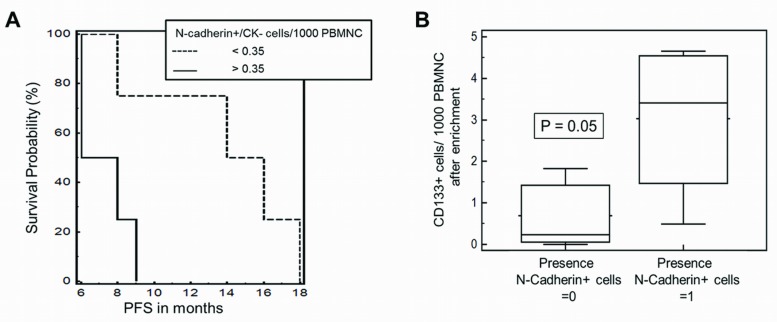
CTC subtypes were associated to clinical outcome. A) Kaplan-Meier test showed that the number of N-cadherin+/CK- cells was significantly associated to progression free survival (PFS) of mRCC patients during first-line treatment with anti-angiogenesis therapy (PFS; 7 vs. 15 months; p = 0.03; [HR] = 0.31; CI: 0.06–1.59). B) Mann-Whitney test revealed a significantly increased number of CD133+ cells in the presence of N-cadherin+ cells (p = 0.05).

Recursive descent partition analysis was used to identify associations of CTC subgroups and outcome related data. The number of mesenchymal N-cadherin+/CK- cells and the amount of CD133+ cells showed a high correlation with response and progression free survival (PFS). The Presence of N-cadherin+/CK- cells was significantly, but inversely correlated to PFS (p = 0.04; r = -0.59). Mann-Whitney test showed that the number of N-cadherin+/CK- was significantly lower in responders vs. non-responders (p = 0.05). Kaplan-Meier log-rank test revealed that an increased number of N-cadherin+/CK- cells (>0.35) significantly correlated to shortened PFS (7 vs. 15 months; p = 0.03; [HR] = 0.31; CI: 0.06–1.59; Fig 4A).

The number of CD133+ cells was significantly associated to the presence of N-cadherin+ cells (p = 0.04), the total amount of N-cadherin+ cells (p = 0.02) and the number of N-cadherin+/CK+ cells (p = 0.04). The ratio of N-cadherin+ cells to CK+ cells (mesenchymal to epithelial cell type) increased significantly when CD133+ cells were present (p = 0.05). Accordingly, we detected a significantly increased number of N-cadherin+ cells in the presence of CD133+ cells (p = 0.004) and in turn a significantly increased number of CD133+ cells in the presence of N-cadherin+ cells (p = 0.05, Fig 4B**)**.

### Gene expression analysis and correlation to cell subtypes

Gene expression levels of candidate genes were measured in the EpCAM-enriched (CD45-/EpCAM+) and in the remaining CD45- and EpCAM-depleted (CD45-/EpCAM-) fraction. Gene expression levels were correlated with clinical parameters, among each other and compared between the CD45-/EpCAM- and the CD45-/EpCAM+ fraction to test for genes that may be independent from CTC subgroups. The *FGFR1* mRNA expression was significantly increased in the CD45-/EpCAM- fraction in relation to the CD45-/EpCAM+ fraction (p = 0.03). *VEGFA* expression was significantly increased in the CD45-/EpCAM+ fraction compared to the CD45-/EpCAM- fraction (p = 0.004).

**Fig 5:**
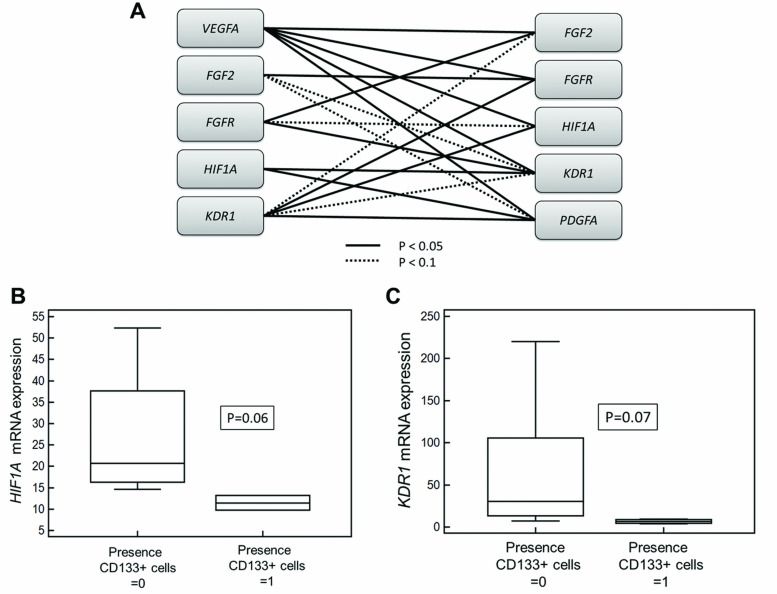
Gene expression analysis. A) Visualization of significant interrelationships between gene expressions. B and C) Mann-Whitney test showed that mRNA expression levels of *HIF1A* (B) and *KDR1* (*VEGFR)* (C) were significantly decreased (p = 0.05) in the EpCAM-/CD45- fraction when CD133+ cells were present in the blood sample.

There were no significant differences of *PDGFA-*, *VEGFR-*, *HIF1A* -and *FGF2* gene expression levels between the both cell fractions. Expression levels of *HIF1A* (p = 0.05, r = 0.56), *PDGFA* (p = 0.01, r = 0.67) and *VEGFR* (p = 0.01, r = 0.71) were significantly correlated to LDH. *FGFR1* mRNA expression in the CD45-/EpCAM- fraction was significantly correlated to response (p = 0.001; r = 0.72). *VEGFA* was significantly associated to *FGFR1*, *HIF1A*, *KDR1* (*VEGFR)*, *PDGFA* and *FGF2*. Fig 5A depicts the correlation between gene expression levels. *HIF1A* and KDR1 (*VEGFR)* mRNA expression levels were by trend decreased (p = 0.06 and p = 0.07) in the EpCAM-/CD45-depleted fraction when CD133+ cells were present in the blood sample (Fig 5B and 5C).

## Discussion

The aim of this feasibility study was to scrutinize whether our previously established method for detecting CTC subgroups is feasible in mRCC. MPIM-based morphological analysis revealed a variety of CTC subtypes with epithelial, mesenchymal, stem cell-like or mixed characteristics such as N-cadherin+/CK-/CD45-; N-cadherin-/CK+/CD45-; CD133+/CK+/CD45+ and CD133-/CK+/CD45 (low) cells. Analyses of individual CTC profiles indicated that the presence as well as the number of mesenchymal and stem cell-like CTC was associated to poor treatment response. The presence of stem cell-like CD133+ cells and the presence of mesenchymal N-cadherin+/CK- cells were correlated to a shortened PFS. If CD133+ cells were detectable, N-cadherin+/CK- cells were likely to be found. Due to technical limitations (staining on 2 slides) it is impossible to determine whether the close association between N-Cadherin+ and CD133+ cells is related to co-expression on the same cell or to different cells. However, it seems to indicate a link between cells with mesenchymal and stem cell-like characteristics implying both as markers of poor prognosis. Cancer stem cells (CSC) and EMT-type cells are believed to play critical roles in drug resistance and cancer metastasis. The formation of CSC and the event of EMT is a dynamic process which is triggered by the interaction of various cellular signaling pathways such as Hedgehog, Notch, PDGF, Wnt, TGF-β, Akt, and NF-κ B signaling pathways [24]. Also in other entities such as lung [25] and pancreatic cancer [26] CSC have been defined by CD133 expression. Recently, Jiang found that some stem cell markers like CD133 were expressed during EMT of tubular cells in vitro [27]. Mani and colleagues described a link between EMT and stemness states in breast cancer models [28]. Their results illustrated a direct connection of less differentiated stem cells with the mesenchymal-appearing cells generated by EMT. They revealed that cells, that have undergone EMT, behaved similar to stem cells from normal or neoplastic cell populations. A study by Armstrong and colleagues showed that more than 80% of CTC in patients with metastatic castration-resistant prostate cancer co-expressed epithelial proteins such as EpCAM and CK with mesenchymal proteins including N-cadherin and the stem cell marker CD133 [29]. Furthermore, they found that more than 75% of CTC from women with metastatic breast cancer co-expressed CK, vimentin and N-cadherin. Nakajima and colleagues investigated the expression level of EMT markers such as N-cadherin, E-cadherin and vimentin in pancreatic primary and metastatic tumors. They reported a correlation of N-cadherin expression with neural invasion and histological type [30]. Only few reports have been published on CTC in mRCC patients. In a study by Bluemke *et al*. prognostic significance of CTC in mRCC patients was evaluated using density and immunomagnetic enrichment as well as CK 8 and 18 for CTC detection. They found two different CTC populations. One was CK 8/18-positive the other one was CK-negative and hematopoietic lineage-negative, but with tumor like morphology. Cell numbers correlated with the presence of lymph node and distant metastases [7]. Another study using the CellSearch system reported the presence of a CTC population with atypical characteristics and a peculiar gene expression profile, characterized by lack of cytokeratin expression and gain of CD44low expression [8]. For most epithelial tumors progression towards malignancy is accompanied by a loss of epithelial differentiation and a shift towards the mesenchymal phenotype, leading to enhanced cancer cell migration and invasion [31]. According to Gradilone and colleagues, who characterized CTC for CK and markers of EMT, the gain of mesenchymal markers in CTC is correlated to patient prognosis [32,33]. Their data showed that the presence of mesenchymal markers on CTC more accurately predicted a poor prognosis than the expression of CK alone. Here, we confirmed that the presence of mesenchymal-like cells correlates to survival in mRCC patients.

Recently, the existence of circulating mesenchymal stem cells (MSC) derived from peripheral blood was reported [34–36]. It was described that MSC have the ability to migrate from bone marrow to damaged tissue via the circulating peripheral blood to promote regeneration [37]. This process may involve hyper stimulation of bone marrow production using granulocyte colony stimulating factor (G-CSF) resulting in the occurrence of a mixture of MSC, hematopoetic stem cells and other immature progenitor cells [35,38,39]. Furthermore, the literature revealed that MSC migrate to and proliferate within tumor sites [40]. In this study we were able to observe a distinct proportion of cells that stained positive for pan-CK and CD45, a phenomenon already described by Yu and colleagues [41]. The additional CD45+ staining may not be exclusive for hematopoietic cells, but may hypothetically be acquired during the dormant state in the bone marrow or through effects comparable to trogocytosis, i.e. transfer of membrane proteins [42]. Even though this hypothesis cannot be scrutinized by the data at hand it may warrant waiving any depletion of CD45-positive cells as this approach might lead to a loss of cells of interest. However, we took only CD133+/CD45- cells into account during the abovementioned analyses of CTC profiles with stem cell-like characteristics.

Interestingly, *HIF1A*, *KDR1 (VEGFR)* and *VEGFA* expression levels were decreased in the CD45-/EpCAM- fraction in the presence of CD133+ cells with the latter appearing to be a marker of poor outcome. These findings are in line with a study by Chen and colleagues who used immunohistochemistry and described that high expression of CD133 was positively associated with tumor invasion depth, presence of distant metastasis, advanced TNM stage and shorter survival in patients with gastric carcinoma [43] Also other markers, such as circulating entothelial cells (CEC) which were shown to correlate with vascular damage, were investigated, recently. Gruenwald *et al* found that in RCC sunitinib treatment was associated with an early increase of CECs in patients with a prolonged PFS [44]. Due to the fact that CECs express CD45 and CD133, using our enrichment strategy, they would remain in the CD45-depleted and EpCAM-depleted cell fraction. It is highly likely that mRNA expression was measured not exclusively in CTC, but also in CEC and other remaining populations such as hematopoietic cells. To be correct, our analysis should be referred to as profiling of non-hematopoietic cells rather than CTC. Additional markers and elaborated molecular characterization are required to ensure that the mRNA expression profiling reflects CTC gene expression instead of host response. Recently, the development of a novel, OB-cadherin-based method to capture mesenchymal CTC was reported [45] and might be relevant for future analysis.

Taken together, our results support the examination of individual CTC profiles during systemic treatment of metastasized renal cell carcinoma to identify new biomarkers for response and outcome monitoring.

## Supporting Information

S1 Fig Flowcytometric immunophenotyping. Flowcytometric analysis and validation of the CD133 cell surface expression on K562 cells engineered to express the CD133 splice variant s1 encoded by an IRES-eGFP expression cassette (CD133:IEG). Cells were stained with PE-conjugated isotype control or anti-CD133 antibodies.
(TIF)
