# Supplementary figures and images for: Circulating Tumor Cell Composition in Renal Cell Carcinoma
Source: PLoS One. 2016 Apr 21;11(4):e0153018. doi: 10.1371/journal.pone.0153018 (PMC4839694; doi:10.1371/journal.pone.0153018)

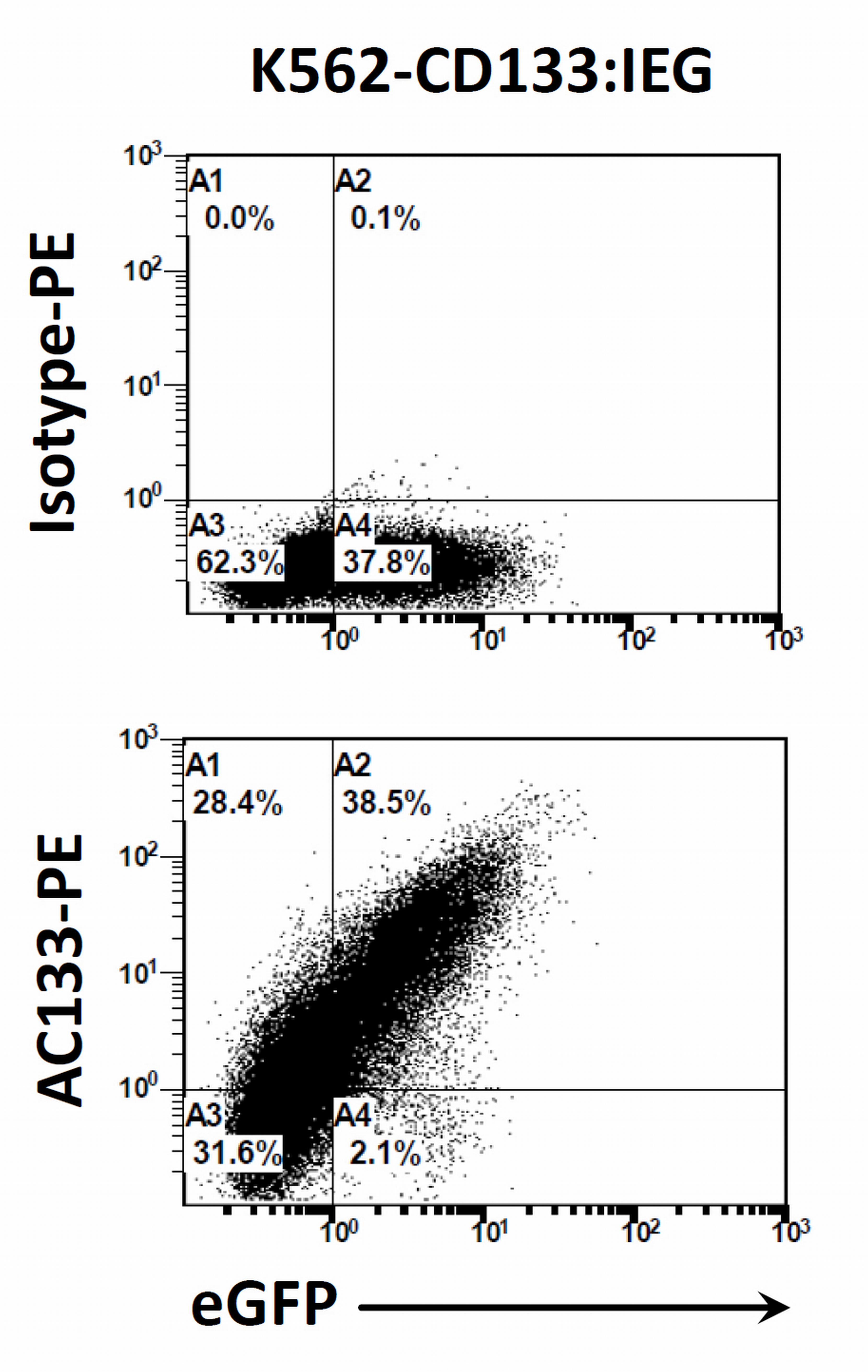

Supplement: S1 Fig — Flowcytometric analysis and validation of the CD133 cell surface expression on K562 cells engineered to express the CD133 splice variant s1 encoded by an IRES-eGFP expression cassette (CD133:IEG). Cells were stained with PE-conjugated isotype control or anti-CD133 antibodies. (TIF) [file pone.0153018.s001.tif]
